# Native and exotic plants play different roles in urban pollination networks across seasons

**DOI:** 10.1007/s00442-023-05324-x

**Published:** 2023-01-24

**Authors:** Vincent Zaninotto, Elisa Thebault, Isabelle Dajoz

**Affiliations:** 1 Institute of Ecology and Environmental Sciences-Paris (iEES-Paris), Sorbonne Université, CNRS, IRD, INRAE, Université Paris Cité, UPEC. 4 Place Jussieu, 75005 Paris, France; 2 Direction des Espaces Verts et de L’Environnement, Ville de Paris, 103 Avenue de France, 75013 Paris, France

**Keywords:** Green spaces, Nestedness, Specialization, Phenology, Invasive species

## Abstract

Urban areas often host exotic plant species, whether managed or spontaneous. These plants are suspected of affecting pollinator diversity and the structure of pollination networks. However, in dense cityscapes, exotic plants also provide additional flower resources during periods of scarcity, and the consequences for the seasonal dynamics of networks still need to be investigated. For two consecutive years, we monitored monthly plant–pollinator networks in 12 green spaces in Paris, France. We focused on seasonal variations in the availability and attractiveness of flower resources, comparing native and exotic plants at both the species and community levels. We also considered their respective contributions to network properties over time (specialization and nestedness). Exotic plants provided more abundant and diverse flower resources than native plants, especially from late summer on. However, native plants received more visits and attracted more pollinator species at the community level; and during certain times of the year at the species level as well. Exotic plants were involved in more generalist interactions, increasingly so over the seasons. In addition, they contributed more to network nestedness than native plants. These results show that exotic plants are major components of plant–pollinator interactions in a dense urban landscape, even though they are less attractive than natives. They constitute a core of generalist interactions that increase nestedness and can participate in the overall stability of the network. However, most exotic species were seldom visited by insects. Pollinator communities may benefit from including more native species when managing urban green spaces.

**Supplementary Information:**

The online version contains supplementary material available at 10.1007/s00442-023-05324-x.

## Introduction

Despite the negative impacts of urbanization on biodiversity, there is evidence that cities can sustain fairly rich pollinator communities, most notably regarding bees (Hall et al. 2017). In particular, Baldock et al. (2019) emphasize the major importance of private gardens and allotments in supporting pollinators, compared to parks and other public green spaces. Nevertheless, in densely populated cities like Paris, private gardens and allotments are scarce, while parks cover larger areas (Shwartz et al. 2013)*.* In such cities, public greenspace management practices are critical to maintaining pollinator biodiversity; and it is necessary to understand which ones best promote plant–pollinator interactions (Mata et al. 2021).

In private gardens and urban parks, much of the available flower resources are provided by ornamental plants, either native or exotic, that are highly variable in their attractiveness to pollinators (Garbuzov and Ratnieks 2014; Garbuzov et al. 2017; Erickson et al. 2020). The geographic origin of these garden plant species raises concerns, since exotic plants can affect pollinator community composition (Pardee and Philpott 2014; Threlfall et al. 2015), whereas native plants are key elements to sustaining rich and functionally diverse insect communities (Mata et al. 2021; Cecala and Wilson Rankin 2021). Yet, the relative contribution of native and exotic plants to urban pollinator communities remains debated (Majewska and Altizer 2020). Exotic flowering plants contribute substantially to the supply of nectar and pollen in urban landscapes (Tew et al. 2021; Casanelles-Abella et al. 2022), thus potentially supporting pollinator communities by increasing overall resources (Tasker et al. 2020; Staab et al. 2020). Indeed, while at the plant community level natives might receive more visits, at the species level some exotics can be very attractive (Lowenstein et al. 2019; da Rocha-Filho et al. 2021). In addition, the relative attractiveness of these plants to pollinators may depend on urbanization levels, due to possible effects of urban environmental stressors on pollinator foraging choices (Buchholz and Kowarik 2019); and most of the mentioned studies were conducted in private gardens, allotments, and nurseries. This issue has yet to be assessed in the context of public green spaces in densely urbanized landscapes*.*

Furthermore, studies rarely explore how exotic garden plants integrate into pollination networks and affect their structure. Yet, network structure is essential in maintaining stability against disturbance (Thebault and Fontaine 2010). Since they can quantify single-species levels of specialization (Blüthgen et al. 2006), network approaches can also help reconcile the contradictory levels of attractiveness observed at community and species levels for exotic plants. Such differences in plant specialization may rely on pollinator preferences (Salisbury et al. 2015), as exotic plants often fail to appeal to specialist pollinator species (Erickson et al. 2020). Looking at invasive plants, some studies have shown that these species often successfully integrate pollination networks, occupying a central place therein (Vilà et al. 2009; Thompson and Knight 2018). The consequences on insect communities vary greatly depending on the context (Stout and Tiedeken 2017; Davis et al. 2018). Invasive plants tend to attract more generalist pollinator species, while specialist pollinators are more strictly dependent on native plants (Parra-Tabla and Arceo-Gómez 2021). As a result, invasive plants display different species-level properties in networks compared to native plants (Arroyo-Correa et al. 2020). They have been found to generate less-specialized pollination networks (Seitz et al. 2020) and create profound topological changes in interactions (Albrecht et al. 2014; Larson et al. 2016). Invasive plants act as super-generalists, notably raising network nestedness (Bartomeus et al. 2008; Russo et al. 2019). In an urban context, it is important to determine whether these results could be applied to exotic garden plants, and how that would affect the structure of pollination networks.

Recent works on plant and pollinator communities emphasize the importance of seasonal dynamics on pollination network structure and species persistence (Guzman et al. 2021). Considering the respective phenologies of plants and pollinators, it is essential to examine interactions from a month-to-month perspective to assess short-term variations in network properties (CaraDonna and Waser 2020). Interestingly, the seasonal dynamics of exotic plants in pollination networks may be different from those of native plants (Larson et al. 2016; Arroyo-Correa et al. 2020; Seitz et al. 2020). Typically, exotic flora has been shown to complement native flora by providing resources for pollinators from late summer on (Salisbury et al. 2015; Staab et al. 2020). While native floral cover fluctuates over time, exotic plants are often selected for their extended and complementary flowering, which can be kept constant by gardening practices (Erickson et al. 2020, 2021). However, these results still need to be confirmed in high-density city contexts where species phenologies can be broader and seasonality less marked than in natural landscapes (Uchida et al. 2018; Zaninotto et al. 2020). There, the seasonal dynamics of pollination networks and their impacts on pollinator diversity are still poorly understood.

Here, we present the results of a 2 year replicated monitoring of insect pollinator activity in the green spaces of a densely urbanized landscape: the city of Paris (France). We investigated plant–pollinator interactions both at the plant species level and the plant community level. We examined pollination networks every month from March to October, assessing the respective roles of the native and exotic floras. We addressed the following questions: (1) How attractive are exotic and native floras to native pollinators, both at the plant community and plant species level? Based on the literature, we hypothesize that native plants attract more native pollinators in general, with wide variation among plant species. (2) How does pollinator visitation of these plants vary over the seasons? We expect exotic plants, at the species and community level, to be visited more often from late summer on. (3) How do these species fit into pollination networks and what are the implications for seasonal network dynamics? We expect exotic plants to be more generalist and contribute more to network nestedness than native plants, leading to more nested and generalist networks from late summer on.

## Methods

### Site location

We selected 12 sampling sites located across the city of Paris (France), at least 1 km apart from each other (average distance to the nearest site: 1902 m ± 170 m SE). Sampling sites were set in pesticide-free green spaces of varying sizes (from 7245 to 161,540 m^2^) and management practices, leading to distinct plant communities. In particular, intensively managed green spaces hosted abundant and diverse garden plant species. In contrast, lightly managed areas contained a majority of spontaneous native plants. More details on sampling sites can be found in Table S1 (ESM).

### Insect sampling

Surveys were conducted at each site for two consecutive years (2019 and 2020), every month from March through October (April 2020 was skipped due to the COVID-19 crisis). All surveys were conducted by the same team, between the 1st and 15th of each month, in alternating order, covering two sites per day. Surveys were only done under conditions favorable to insects (no rain, low wind, temperature > 10 °C), between 8:00 and 15:00 (local solar time).

An active sampling of foraging insect pollinators was conducted along 50 m transects in each site. Sampling was stratified across three habitats: shrubs, lawns, and flowerbeds, based on the respective proportions of these habitats in each green space. Transects were walked twice with a 10 min interval in between, at a slow pace, and with no time limit. All flowers within 1 m on either side of the transect were examined for flower visitors. All flower visitors observed in contact with the fertile parts of a flower were collected with insect nets or plastic boxes, while visited plants were identified to species level. Unambiguously identifiable insects were recorded and released at the end of the survey. The others were euthanized with ethyl acetate vapors and then returned to the laboratory for identification. We identified them at the genus level before sending them to several specialists for identification at the species level. All the preserved specimens are now part of the iEES-Paris laboratory collection (4 place Jussieu, 75005 Paris, France).

### Plant inventories

We conducted monthly plant inventories during each survey, on five 1 × 1 m quadrats evenly distributed over each transect. We identified all flowering plant species in these quadrats and counted the floral units for each species (one floral unit = one individual flower; Apiaceae umbels and Asteraceae flower heads counting as one).

In urban settings, exotic plants encompass both planted garden species and spontaneous species. Depending on their population dynamics, the latter can be classified as subspontaneous, naturalized, or invasive, although this varies over time and can be difficult to differentiate (Richardson et al. 2011)*.* For this reason, we only considered the geographical origin of plant species, classifying them as either ‘native’ or ‘exotic’ (Table S5, ESM). ‘Native’ plants comprised plants originating from the Ile-de-France bioregion, as well as anciently naturalized plants with stable populations (archaeophytes) (Jauzein and Nawrot 2011): 24.6% were either annual or biennial species, and 75.4% were perennial species. ‘Exotic’ plant species included exotic garden plants, recently naturalized plants, but also horticultural varieties, and species with regional invasive status (Wegnez 2018): 24.8% were either annual or biennial species, and 75.2% were perennial species. In the end, most planted garden species (80%) were considered exotic, whereas a majority of spontaneous and subspontaneous plant species (83%) were native.

### Plant community- and species-level measurements

At the plant community level, we described flower availability separately for native and exotic plants in each site, using two indices. First, we calculated monthly values of flower density per m^2^ to represent resource supply, using surveys of the five 1 × 1 m quadrats along each transect. Second, we determined flowering plant species richness per month, along each entire transect, as a proxy of floral diversity. We then assessed the attractiveness of these floral assemblages at the community level by looking at patterns of pollinator visitation over time; this was done by considering the number of interactions and the species richness of interacting pollinators.

At the plant species level, we also investigated pollinator visitation and the structure of mutualistic interactions. For each site and each month, we calculated indices of network structure at the plant species level: the number of interactions, the number of interacting pollinator species (degree), Blüthgen’s index of specialization d’ (Blüthgen et al. 2006), and the species contribution to network nestedness (based on the NODF estimator, Almeida-Neto et al. 2008).

### Statistical analysis

All data analyses were performed with R software version 4.0.5 (R core team 2021). We modeled seasonal variations in floral resource availability and pollinator visitation patterns as a function of the time of year. To this end, we considered time as a continuous variable (day of the year), using a degree 2 polynomial, since we expected unimodal seasonal patterns (Fig. 2 and 3). In addition, we built models with time as a factor (month), to conduct post hoc mean comparisons between exotic and native plants at specific times (emmeans package, Lenth 2019) (Fig. S2 & Table S4, ESM).

At the community level, we constructed generalized linear mixed models (GLMM) of flower resources over time, with negative binomial error distributions and zero inflation. We modeled flower density (number of floral units per m^2^) and floral species richness (total number of plant species in bloom per site) (Fig. 2 and Table 1). Fixed effects were the plant origin (‘native’ or ‘exotic’), the day of the year (degree two polynomial) and their interaction, green space size, and the year (2019 or 2020); the site was included as a random effect (*n* = 12).

**Table 1:** GLMM of the seasonal variations in flower resources (flower density and floral species richness), and their attractiveness to pollinators (number of interactions; number of pollinator species) at the community level, for native and exotic plants

Response variable and predictors	*χ*^2^	*P*	Estimates
Flower density			
(Negative binomial, AICc = 3920, *r*^2^ = 0.39)			
Day^2^	32.9 (2df)	7.4e−08	See Fig. 2
Origin	9.5 (1df)	0.0020	See Fig. 2
Day^2^ × origin	30.0 (2df)	3.1e−07	See Fig. 2
Year	18.1 (1df)	2.1e−05	5.4 ± 0.2 (2019), 4.7 ± 0.2 (2020)
Floral species richness			
(Negative binomial, AICc = 1785, *r*^2^ = 0.28)			
Day^2^	69.4 (2df)	8.6e−16	See Fig. 2
Origin	39.2 (1df)	3.8e−10	See Fig. 2
Day^2^ × origin	31.6 (2df)	1.4e−07	See Fig. 2
Number of interactions			
(Quasi-Poisson, AICc = 1963, *r*^2^ = 0.77)			
Day^2^	63.3 (2df)	1.8e−14	See Fig. 2
Origin	44.2 (1df)	3.0e−11	See Fig. 2
Flower density (log)	101.9 (1df)	< 2.2e−16	Slope: 0.59 ± 0.06
Floral species richness	135.0 (1df)	< 2.2e−16	Slope: 0.53 ± 0.05
Green space size	11.8 (1df)	0.00060	Slope: 0.17 ± 0.05
Pollinator richness			
(Quasi-Poisson, AICc = 1456, *r*^2^ = 0.70)			
Day^2^	59.3 (2df)	1.3e−13	See Fig. 2
Origin	47.1 (1df)	6.9e−12	See Fig. 2
Flower density (log)	42.8 (1df)	6.0e−11	Slope: 0.34 ± 0.05
Floral species richness	149.0 (1df)	< 2.2e−16	Slope: 0.50 ± 0.04
Green space size	11.7 (1df)	0.00062	Slope: 0.14 ± 0.04

The predictors are given after variable selection. *χ*^2^ and associated *P* values give the results of Type-III Wald analysis of deviance; df: degrees of freedom of the *χ*^2^ test

*AICc* Second-order Akaike Information Criterion, *r*^2^ is the conditional r-squared value considering both the fixed and random effects

Then, we considered patterns of pollinator visitation at the plant community level, via GLMM with Quasi-Poisson error distributions and zero inflation. We thus modeled the number of interactions and the number of pollinator species per site and month (Fig. 2 and Table 1). Again, fixed effects were the plant origin (‘native’ or ‘exotic’), the day of the year (degree two polynomial) and their interaction, green space size, and the year (2019 or 2020), to which we added flower resources (log-transformed flower density and floral species richness per site and month). This allows us to account for the confounding effect of resource diversity and abundance along transects on pollinator visitation. The site was included as a random effect (*n* = 12).

**Fig. 1:**
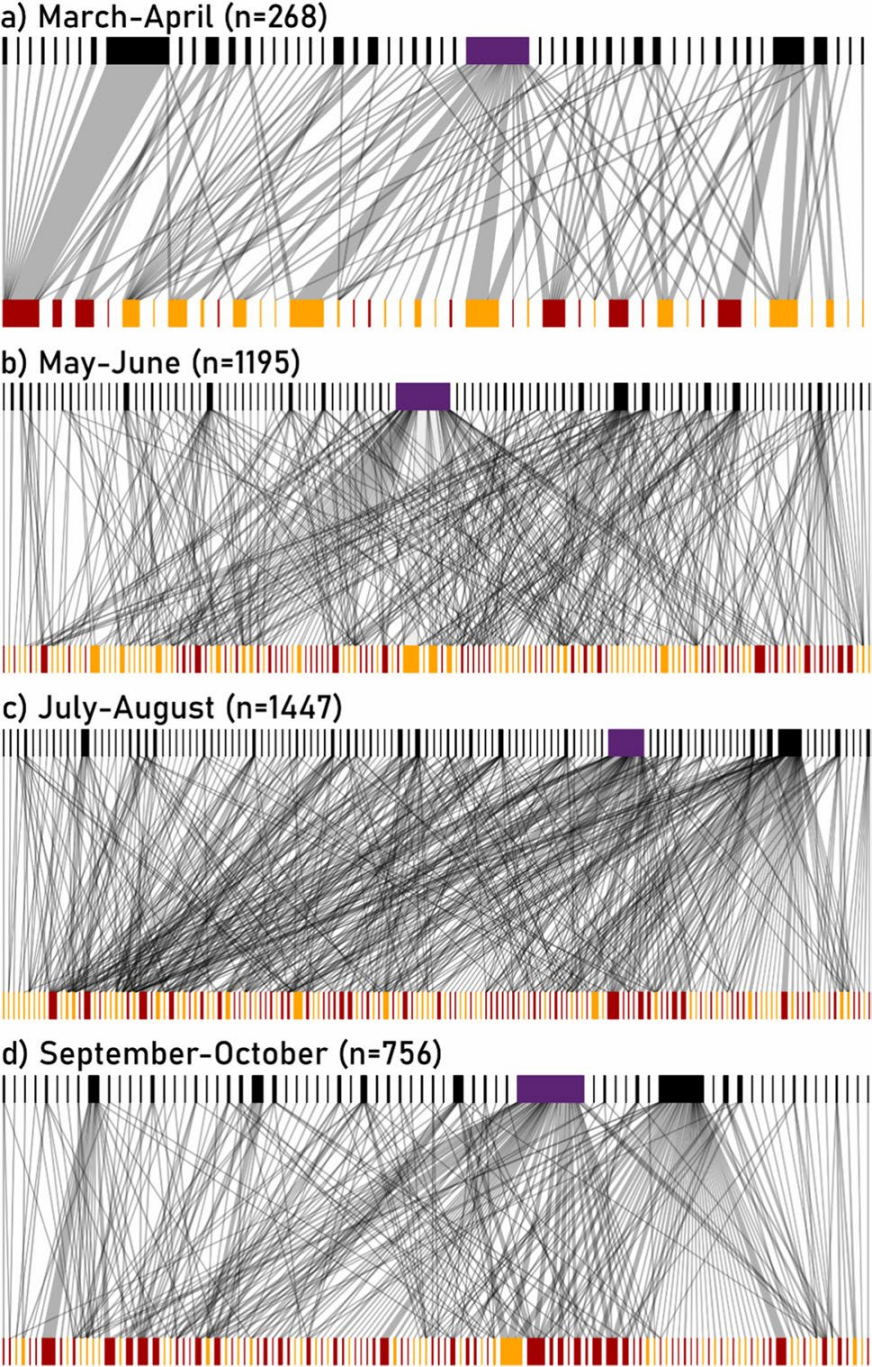
Representation of the bipartite plant–pollinator networks over seasons. Data are pooled across all sites and per 2-month period. Upper boxes represent pollinator species, and width is proportional to the number of interactions performed by each species (n: total number of interactions per period) (purple box: *Apis mellifera*, black boxes: other species). Lower boxes represent plant species according to their origin (orange: native, red: exotic), and width is proportional to each plant species' flower density (log-transformed) during each period. Gray links represent interactions

Besides, we investigated interaction networks at the community level, using the bipartite package (Dormann et al. 2008). We modeled the seasonal variations of the network-level nestedness (NODF) and specialization (H2’, the network-level equivalent to *d*’) (Fig. S1 & Table S2, ESM). Fixed effects included overall flower density and network size, known to influence network structure, as well as the day of the year (degree two polynomial), green space size, and the year (2019 or 2020); the site was included as a random effect (*n* = 12). We plotted networks aggregated over 2 months and all 12 sites, for visual clarity (Fig. 1).

At the plant species level, we also used GLMM to study the seasonal variations of patterns of pollinator visitation: we modeled the number of interactions per plant species, and the degree (number of interacting pollinators) per site and month, with Poisson error distributions and zero inflation (Fig. 3 and Table 2). Fixed effects included: the plant origin (‘native’ or ‘exotic’), the day of the year (degree two polynomial) and their interaction, the plant growth form (‘annual/biennial’ or ‘perennial’), flower density per plant species and month, the year (2019 or 2020), and green space size; the plant species (*n* = 346) and site (*n* = 12) were considered as random effects.

**Table 2:** GLMM of the seasonal variations in plant–pollinator interactions structure at the plant species level (number of interactions, degree, contribution to nestedness, and specialization index *d*’), for native and exotic plants

Response variable and predictors	*χ*^2^	*P*	Estimates
Number of interactions per plant			
(Poisson, AICc = 6520, *r*^2^ = 0.52)			
Day^2^	116.5 (2df)	< 2.2e−16	See Fig. 3
Origin	5.5 (1df)	0.019	See Fig. 3
Day^2^ × origin	5.0 (2df)	0.082 (NS)	See Fig. 3
Flower density/species (log)	312.9 (1df)	< 2.2e−16	Slope: 0.37 ± 0.02
Year	6.7 (1df)	0.0094	0.6 ± 0.1 (2019), 0.5 ± 0.1 (2020)
Pollinator richness per plant (degree)			
(Poisson, AICc = 4741, *r*^2^ = 0.43)			
Day^2^	74.4 (2df)	< 2.2e−16	See Fig. 3
Origin	4.9 (1df)	0.027	See Fig. 3
Day^2^ x origin	8.0 (2df)	0.019	See Fig. 3
Flower density/species (log)	63.6 (1df)	1.5e−15	Slope: 0.21 ± 0.03
Contribution to nestedness			
(Gaussian, AICc = 2218, *r*^2^ = 0.19)			
Day^2^	22.3 (2df)	1.5e−05	See Fig. 3
Origin	4.6 (1df)	0.031	See Fig. 3
Flower density/species (log)	4.5 (1df)	0.034	Slope: 0.06 ± 0.03
Year	9.2 (1df)	0.0025	− 0.3 ± 0.1 (2019), − 0.2 ± 0.1 (2020)
Specialization (*d*’)			
(Beta regression, AICc = -1677, *r*^2^ = 0.67)			
Day	8.7 (1df)	0.0033	See Fig. 3
Origin	12.6 (1df)	0.00039	See Fig. 3
Day × origin	8.0 (1df)	0.0046	See Fig. 3
Year	5.6 (1df)	0.018	0.3 ± 0.1 (2019), 0.1 ± 0.1 (2020)

The predictors are given after variable selection. *χ*^2^ and associated *P* values give the results of Type-III Wald analysis of deviance; *df*: degrees of freedom of the *χ*^2^ test

We also considered network indices at the plant species level: the contribution to nestedness, and the specialization index (*d*’) per site and month (Fig. 3 and Table 2). The species contribution to nestedness (*nestedcontribution* function, bipartite package) followed a continuous distribution centered on zero (positive values representing a positive contribution to nestedness) and was modeled with a Gaussian error distribution. Meanwhile, *d*’ is comprised between 0 and 1 (*specieslevel* function, bipartite package; high values indicating high specialization), and was modeled with a beta-regression. A zero–one-inflated beta-distribution model achieved better normality of residuals but presented very similar results, so we present the simplest model. Fixed effects included: the plant origin (‘native’ or ‘exotic’), the day of the year (degree two polynomial) and their interaction, the plant growth form (‘annual/biennial’ or ‘perennial’), flower density per plant species and month, network size, and the year (2019 or 2020); the plant species (*n* = 346) and site (*n* = 12) were considered as random effects.

For all models, we performed variable selection, picking the best models based on the second-order Akaike Information Criterion (AICc, Barton 2020). We assessed the contributions of explanatory variables through analysis of deviance using Wald type-III chi-square tests (Fox and Weisberg 2019). Normality of residuals and homoscedasticity were verified using DHARMa package (Hartig 2021). For each model, we checked the variables for collinearity using the package performance (Lüdecke et al. 2021, all VIFs were < 1.3). We also performed Moran tests on the residuals of each model and detected no spatial autocorrelation.

## Results

### Overview of plant–pollinator interactions

Over the 2 years and across the 12 sites, we recorded a total of 3666 plant–pollinator interactions. A large majority of the insects were identified at the species level (95.9%) and the remaining at the genus level (full list in Table S6, ESM). They were distributed among four orders: Hymenoptera, Diptera, Lepidoptera, and Coleoptera. Hymenoptera were dominated by wild (non-domesticated) bees, of which we recorded 90 species (accounting for 52.3% of interactions), and managed honey bees (30.2% of interactions); although we also collected non-bee Hymenoptera (16 taxa, 1.2% of interactions). The second most abundant order was Diptera (55 taxa, 12.3% of interactions), including 30 species of hoverflies. Last came Lepidoptera (13 species, 3.7% of interactions), and Coleoptera (4 species, 0.4% of interactions). All but three pollinator species were native to the region, and only 0.7% of interactions were realized by exotic insects.

Meanwhile, we recorded a total of 346 plant species and varieties (Table S5, ESM). The vast majority of species were represented by only one variety or cultivar. Only six garden plant species were observed in two forms: either in normal form or in a form with extra petals. In that case, we treated the two cultivars as different species in the analyses. Of all plant species recorded, 158 were categorized as native (79.8% were visited by insects), and 188 as exotic (75.0% were visited). Exotic plants comprised seven invasive species, five of which were visited by pollinators during our surveys (*Buddleja davidii, Erigeron canadensis*, *Galega officinalis*, *Impatiens balfourii*, and *Senecio inaequidens*) and two that were not (*Berberis aquifolium* and *Erigeron annuus*). Among the 179 insect taxa identified, 40.2% visited only native plant species, 12.3% visited only exotic species, and 47.5% visited both native and exotic species.

### Flower resource availability and attractiveness at the community level

**Fig. 2:**
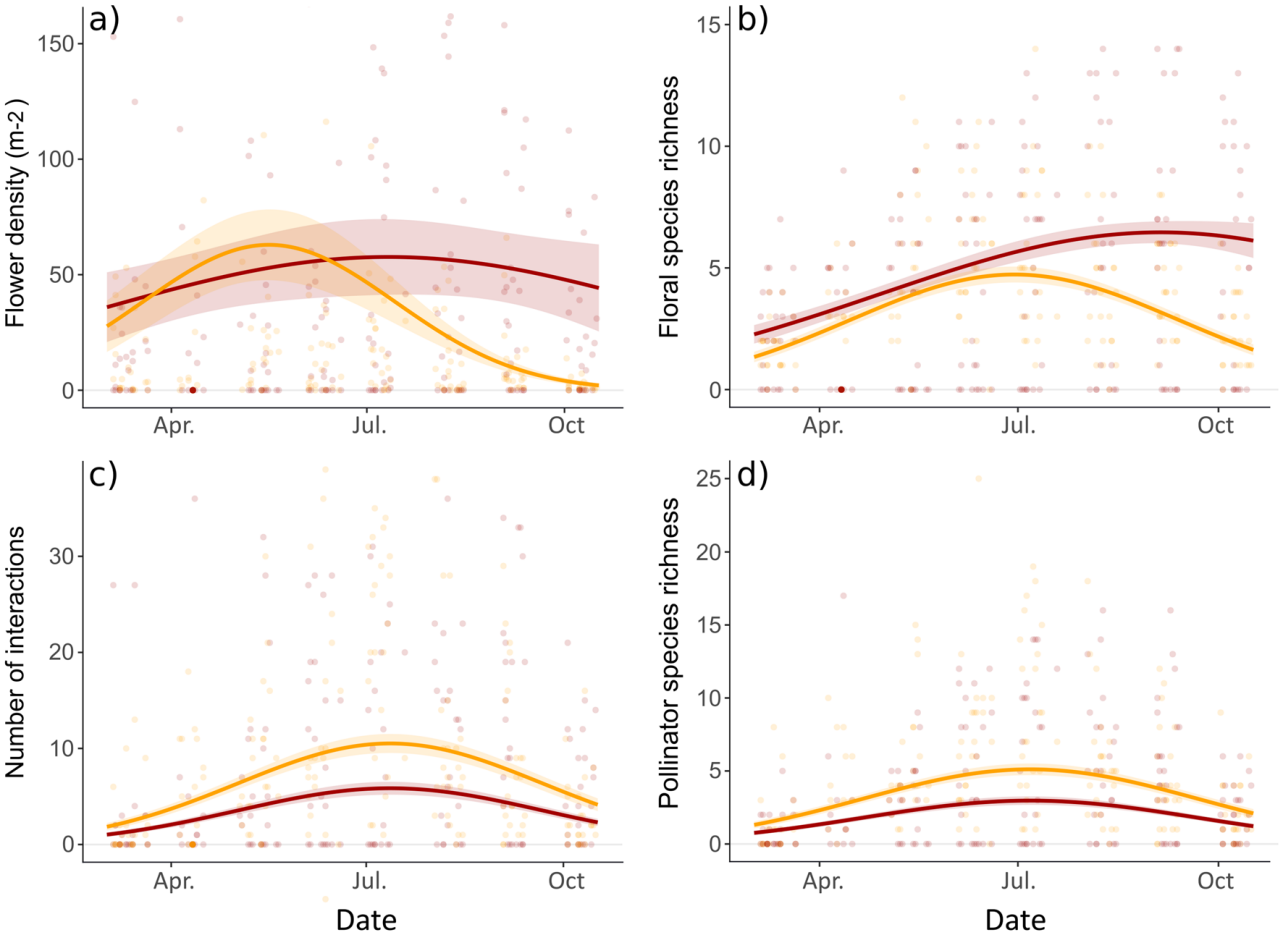
Seasonal variations in flower resources: **a** flower density per m^2^ and **b** floral species richness per site; and their attractiveness to pollinators at the community level: **c** number of interactions and **d** number of pollinator species; for native and exotic plants (native: orange, exotic: red). Lines indicate predictions from the GLMM presented in Table 1 (± SE), and points represent observed values. The **c** number of interactions and **d** pollinator species richness are modeled by accounting for the variations of flower resources

We used flower density and floral species richness as indicators of flower resource availability at the community level, and studied them over time and by origin (Fig. 2). As revealed by the significant interaction “Day^2^ × Origin”, exotic and native plants followed different unimodal seasonal patterns (Table 1), with a 2 month delay in maximal values. Indeed, the peak of flower density came in May for native flora and in July for exotic flora (Fig. 2a), whereas the peak of floral species richness came in July for native flora and in September for exotic flora (Fig. 2b). Overall, the exotic flora displayed significantly more floral units and plant species during August, September, and October (Fig. S2 & Table S4, ESM). Thus, we observe two phases: the first in spring with equivalent resources of both types, and the second from mid-summer onwards when resources provided by exotic plants became dominant (red boxes in Fig. 1).

At the community level, the number of interactions and pollinator species richness per site and month were both strongly and positively related to flower density (respective slopes: 0.59 ± 0.06 and 0.34 ± 0.05, Table 1), to floral species richness (respective slopes: 0.53 ± 0.05, and 0.50 ± 0.04, Table 1), and to green space size (respective slopes: 0.17 ± 0.05 and 0.14 ± 0.04, Table 1). Notably, for a given level of flower resources, the native flora was always more attractive to pollinators than the exotic flora, as the number of interactions and pollinator richness were significantly always higher for natives than for exotics (Fig. S2 & Table S4, ESM). Also, pollinator visitation exhibited unimodal seasonal patterns independent of variation in flower availability. Both types of flora attracted more pollinator individuals and species during summer, with a peak in early July (Fig. 2c, d.). Controlling for flower resources, there was no delay between seasonal patterns of visitation of native and exotic plant species, as the interaction term “Day^2^ × Origin” was not significant. Because of the seasonal increase in interaction abundance and pollinator species richness, but also the rise in plant diversity, we observed a summer increase in network size (Fig. 1).

### Network properties at the plant species level

**Fig. 3:**
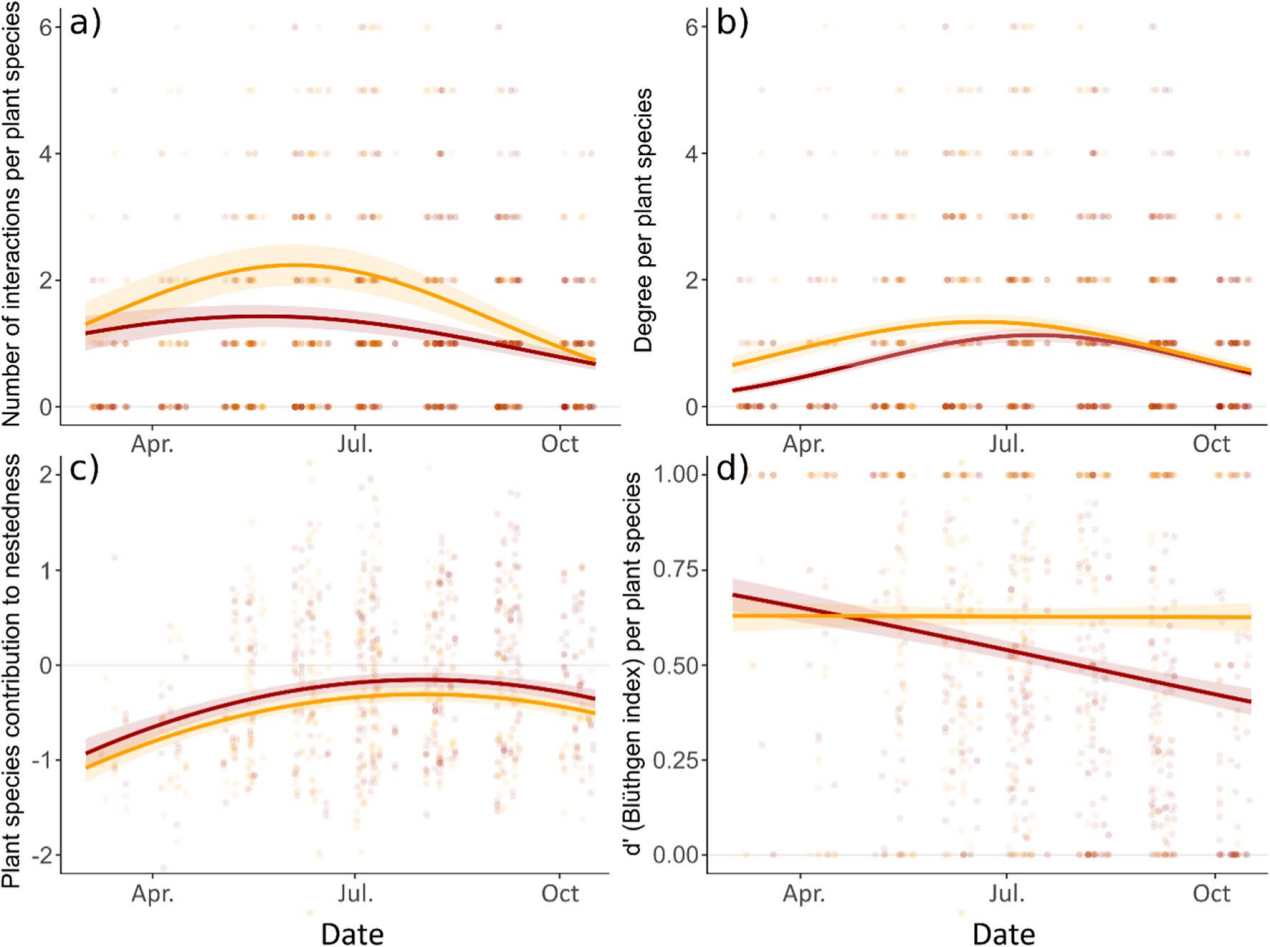
Seasonal variations in plant–pollinator interactions at the plant species level: **a** number of interactions per plant species, **b** number of interacting pollinator species per plant species (degree), **c** contribution to monthly network nestedness per plant species, and **d** specialization index *d*’ per plant species; for native and exotic plants (native: orange, exotic: red). Lines indicate predictions from the GLMM presented in Table 2 (± SE), and points represent observed values. Indices are modeled also accounting for the variations of flower density per plant species

At the plant species level, we also observed significant differences based on plant origin (“exotic” vs. “native”) but not on growth form (“annual/biennial” vs. “perennial”) (Table 2). Pollinator visitation differed by plant origin during the summer (Fig. 3a), with native plant species receiving significantly more visits than exotic ones in June, July, and August (Fig. S2 & Table S4, ESM). Besides, the number of pollinator species (degree) was higher for native plants than for exotic plants in spring and early summer, although the difference was really significant only in March (Fig. 3b, Fig. S2 & Table S4, ESM). The number of interactions per plant species and the degree were also both positively influenced by flower density (respective slopes: 0.37 ± 0.02 and 0.21 ± 0.03, Table 2).

Plant contribution to monthly network nestedness, at the species level, also followed a seasonal pattern with a maximum occurring in summer (late July, Fig. 3c). Although exotic and native plant species followed the same temporal pattern (the interaction effect “Day^2^ × Origin” was not significant), exotic plants overall contributed more to the nestedness than native ones (Table 2, Fig. S2, Table S4, ESM). Meanwhile, network-level nestedness followed a unimodal seasonal pattern with a peak in July, coinciding with the maximum size and diversity of the networks (Fig. S1 & Table S2, ESM).

In addition, we calculated the specialization index (*d*’) for each plant species. For native plants, d’ remained stable over time around a mean value of 0.63. Meanwhile, for exotic plants, d’ decreased over time (Fig. 3d, Table 2), becoming significantly lower than for native plants in August, September, and October (Fig. S2 & Table S4, ESM). Thus, from late summer on, exotic plant species were involved in more generalist interactions than native species, whereas there was no difference between them during spring. Overall network-level specialization (H2’), encompassing both native and exotic flora, similarly decreased over time (Fig. S1 & Table S2, ESM).

## Discussion

Despite providing more flowers than natives in urban green spaces, especially from late summer on, exotic plants were less attractive after controlling for flower abundance and diversity. Also, these plants occupied different positions in urban pollination networks, as exotics contributed more to nestedness than native plants and showed greater generalism levels from August onwards, partly driving the seasonal dynamics of network structure.

### Exotic plants are less attractive than natives but more available from late summer on

Our results bring further support to the importance of native plants for urban pollinators, as found previously in city private gardens (Salisbury et al. 2015; Lowenstein et al. 2019). In the context of a densely populated city, native plants attracted more pollinator individuals and species than exotic plants. This emerged when correcting for flower availability, meaning that natives are more attractive than exotics when equally available. Such a result was obtained at the plant community level, but also at the plant species level, although at this scale, it was limited to specific times of the year (early spring for flower visitor richness and summer for flower visitor abundance). Overall, the preference for natives is illustrated by the large share of pollinator species that visited only these plants (40.2%), while few (12.3%) visited only exotic plants. Given such preferences, promoting native flora in green spaces seems beneficial for pollinator abundance and diversity.

We found that flower density and diversity, as well as green space size, contribute strongly to pollinator richness and abundance, as is typically observed at the community level (Ayers and Rehan 2021, for a review). However, native and exotic flower resources are not equally available in those green spaces, with distinct seasonal dynamics. In particular, starting in August, exotic flowers are more abundant and diverse than native ones. This finding has also been recently reported in private residential gardens (Staab et al. 2020), suggesting that the relative importance of native and exotic plants for pollinators can only be fully understood from a seasonal perspective. The supply of exotic flowers in late summer and fall can be explained by better resistance to summer heat and drought, as well as better maintenance by gardeners; while many native plants decline in bloom by the end of the summer. As a result, exotic plants may be more visited than natives starting in August, as previously reported (Salisbury et al. 2015; Staab et al. 2020). This suggests that exotics may supplement the resources provided by native plants from late summer on, despite being less attractive on their own.

Though the native flora attracted overall more pollinators than the exotic flora, seasonal patterns of plant attractiveness were similar. Pollinators were more abundant and diverse during summer, with a maximum in early July for native and exotic floras alike. This may reflect a general increase in pollinator abundance in the environment at this time of the year (Zaninotto et al. 2020). In contrast, there was a difference in seasonal dynamics at the plant species level, with native plant species attracting more pollinator species than exotic ones during spring*.* This is consistent with the observations of Cecala and Wilson Rankin (2022), with higher bee diversity in native-rich nurseries than in the conventional nurseries, albeit exclusively in spring. Previous studies have shown that early flying bees are more dependent on natural habitats within urban areas (Harrison et al. 2018; Banaszak-Cibicka et al. 2018)*,* possibly relying more on native plants. This would explain why visitors of exotic flowers are less diverse in spring.

### Exotic and native plants affect network structure differently

Our results show that exotic species tend to have a central position in urban pollination networks and that this position varies throughout seasons. Indeed, as we hypothesized, native plant species were involved in more specialized interactions than exotic species. This effect was only detectable starting in August, as exotics displayed decreasing values of the specialization index (d’) over time. Thus, from late summer on, exotic plants attracted visitors in a generalist way, without distinguishing among the available pollinators. This is consistent with evidence that exotic plants attract less-specialized bee species than native plants (Cecala and Wilson Rankin 2021). The observed seasonal trend could be due to higher proportions of generalist pollinators late in the year, taking advantage of the abundant exotic floral resources at this time. In any case, exotic plants appear to drive a general decrease in specialization at the network scale (H2’ index). The urban environment is known to apply filtering to bee traits, among them generalism. As a result, generalist bees are more prominent in cities (Casanelles-Abella et al. 2022), as also demonstrated in Paris (Geslin et al. 2015). Our results suggest that this phenomenon may partly rely on the abundance of exotic plants, which favor generalist pollinators.

As we also hypothesized, exotic plants contributed more to network nestedness than native ones. Exotic plants occupy a central position in the networks, consistent with what has been observed with invasive species (Bartomeus et al. 2008; Larson et al. 2016; Russo et al. 2019)*.* Although a nested structure implies potential competition between generalist and specialist pollinators, it is generally thought to provide a buffer against specialist extinction (Tylianakis et al. 2010). Hence, by increasing nestedness, exotic plants may contribute to network stability, although our knowledge is still insufficient to accurately predict population levels and ecosystem functions from network properties only (Valdovinos 2019). In addition, we noted seasonal dynamics of contribution to nestedness, which were similar for exotics and natives. Interaction networks became bigger in the summer, with a more nested structure. This again underlines the need to consider month-to-month variations in network structure (CaraDonna and Waser 2020). However, like most studies, we constructed our interaction networks based on pollinator foraging behavior. Yet, some interactions bear low value to plant species fitness. When considering the efficiency of pollination interactions, networks can be considerably smaller, with less-connected, generalized, and nested structures (de Santiago-Hernández et al. 2019). The centrality of exotic garden plants in urban pollination networks may likewise not be supported by actual pollen transfers. In fact, the presence of these plants often does not depend on reproductive success, as they are regularly replaced by gardeners.

### Guidelines to greenspace managers

In urban green spaces, pollinator-friendly varieties are frequently planted without regard to species origins. While this is a way to increase flower availability, this practice can lead to the introduction of invasive plants (Johnson et al. 2017), illustrating the potential unintended consequences of garden plants. Meanwhile, similar issues apply to managed pollinator fauna. Indeed, here, nearly one-third of the interactions involved managed honey bees. They visited 71 native and 82 exotic plant species (representing, respectively, 56.3% and 58.2% of visited species). As can be seen in Fig. 1, they are core contributors to Parisian pollination networks. As such, they may enhance network stability, much like exotic plants. However, in Paris, high densities of honey bee hives have been shown to drive a decrease in wild pollinator visitation activity (Ropars et al. 2019). Honeybees could also facilitate the integration of exotic plants into pollination networks, as they visit them abundantly (Urbanowicz et al. 2020; Parra-Tabla and Arceo-Gómez 2021). In return, the dominance of exotic plants in urban green spaces may benefit honey bees but hinder more specialized bees (Threlfall et al. 2015).

In British cities, Baldock et al. (2019) took note of native and exotic plant species that attracted more pollinators than expected based on their flower densities. Here, we recovered some of the plants they recorded as attractive and again found that they were visited abundantly (Table S5, ESM). Our most visited plants, however, were not on their list, and comprised both native and exotic species: e.g., *Helminthotheca echioides* and *Trifolium repens* (native species), *Verbena bonariensis* and *Phacelia tanacetifolia* (exotic species). We recommend planting such pollinator-friendly plant species, with consideration for seasonal successions. As confirmed by our results, flower density and diversity are key to attracting and sustaining pollinators, though it is better to favor plant species that are complementary in both phenology and insect visitor assemblage composition*.* Without being an absolute criterion, the geographical origin of plant species must be taken into consideration when designing green spaces (Buckley and Catford 2016). On the one hand, exotic garden plant species may support more nested networks and provide additional resources for generalist pollinators. On the other hand, native plants attract more pollinators for a given level of flower density and support more diverse pollinator communities. As they are involved in more specialized interactions, they also contribute to functional diversity. While it may be difficult to maintain a high floral density with only native plants, we strongly recommend that these plants be given preference in the design and management of green spaces.

## Supplementary Information

Below is the link to the electronic supplementary material.

Supplementary file1 (PDF 1474 KB)

## Data Availability

Data are available in the publicly accessible repository Zenodo, within the “iEES-Paris OpenData” community (https://doi.org/10.5281/zenodo.7488942).
