# Correction to: Native and exotic plants play different roles in urban pollination networks across seasons

**DOI:** 10.1007/s00442-023-05356-3

**Published:** 2023-03-24

**Authors:** Vincent Zaninotto, Elisa Thebault, Isabelle Dajoz

**Affiliations:** 1 Institute of Ecology and Environmental Sciences‑Paris (iEES‑Paris), Sorbonne Université, CNRS, IRD, INRAE, Université Paris Cité, UPEC. 4 Place Jussieu, 75005 Paris, France; 2 Direction des Espaces Verts et de L’Environnement, Ville de Paris, 103 Avenue de France, 75013 Paris, France

**Correction to:** **Oecologia (2023) 201:525–536 **10.1007/s00442-023-05324-x

The scale of Fig. 2a should be (0-50-100-150) the scale of Fig. 2b should be (0-5-10-15). Revised version of the image is updated here.

**Fig. 2:**
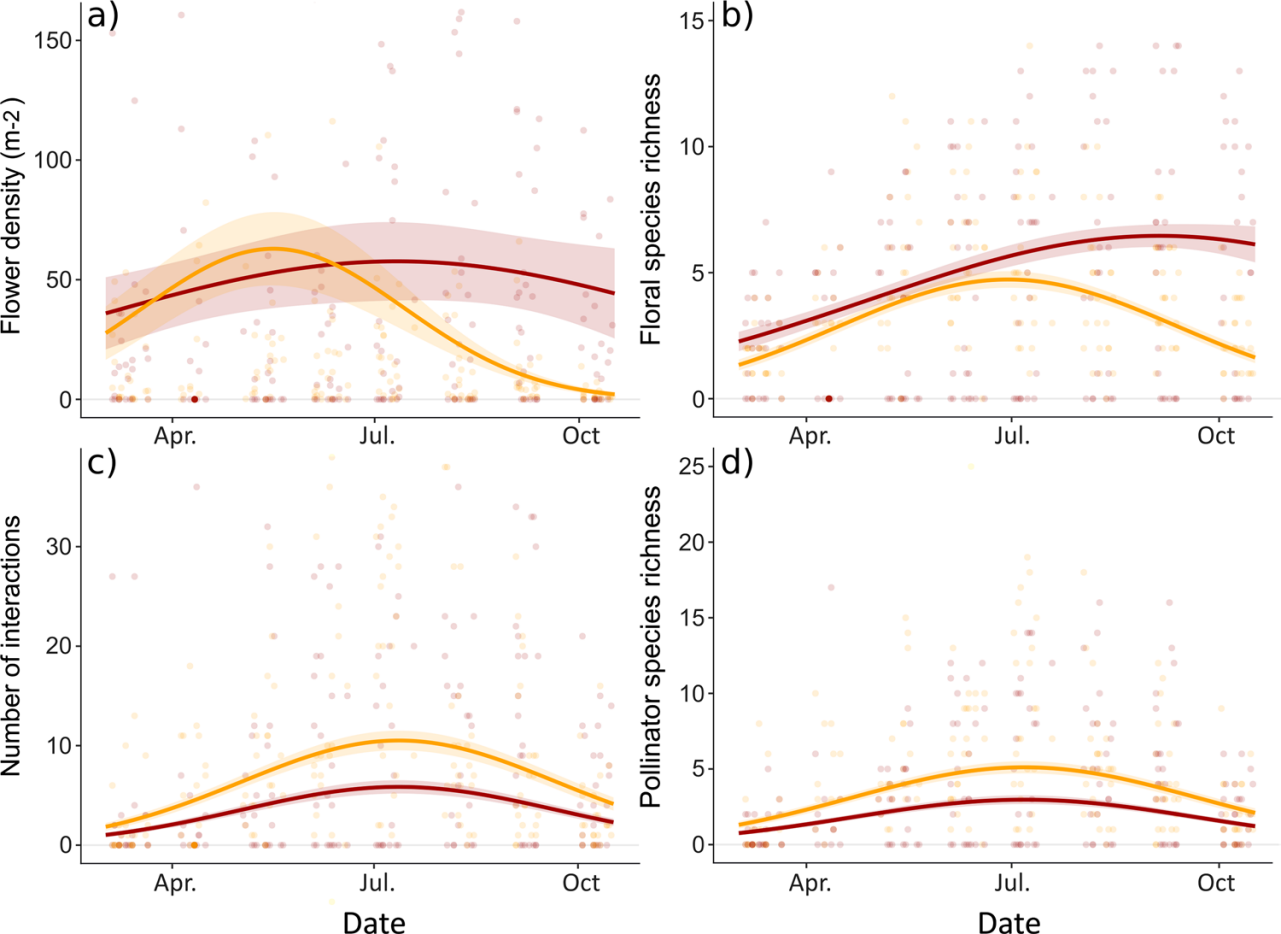
Seasonal variations in flower resources: **a** flower density per m^2^ and **b** floral species richness per site; and their attractiveness to pollinators at the community level: **c** number of interactions and **d** number of pollinator species; for native and exotic plants (native: orange, exotic: red). Lines indicate predictions from the GLMM presented in Table 1 (± SE), and points represent observed values. The **c** number of interactions and **d** pollinator species richness are modeled by accounting for the variations of flower resources

The original article has been corrected.

