# Supplementary material for: Native and exotic plants play different roles in urban pollination networks across seasons
Source: Oecologia. 2023 Jan 24;201(2):525–36. doi: 10.1007/s00442-023-05324-x (PMC9872067; doi:10.1007/s00442-023-05324-x)
Supplement: Supplementary file 1 — Supplementary file1 (PDF 1474 KB) [file 442_2023_5324_MOESM1_ESM.pdf]

Electronic Supplementary Materials

Article title: Native and exotic plants play different roles in urban pollination networks across seasons

in ***Oecologia***

Vincent ZANINOTTO [1,2,*], Elisa THEBAULT[1], Isabelle DAJOZ[1]

1. Institute of Ecology and Environmental Sciences-Paris (iEES-Paris), Sorbonne Université, CNRS, IRD, INRAE, Université Paris Cité, UPEC. 4 place Jussieu, 75005 Paris, France.
2. Direction des Espaces Verts et de l'Environnement, Ville de Paris. 103 avenue de France 75013 Paris, France.

* corresponding author (vincent.zaninotto@normalesup.org) (ORCID: 0000-0002-3397-398X)

CONTENTS

1. **Supplementary Figures**

- Figure S1. Seasonal variations in plant-pollinator interactions properties at network level
- Figure S2. Seasonal variations in flower resource availability and plant-pollinator interactions, with time of year as a discrete variable

2. **Supplementary Tables**

- Table S1. Detailed information on the 12 selected green spaces and their flower resources
- Table S2. GLMM of the network-level seasonal variations in plant-pollinator interaction nestedness and specialization index
- Table S3. GLMM of seasonal variations in flower resource availability and plant-pollinator interactions, with time of year as a discrete variable
- Table S4. Post-hoc comparisons of flower resource availability and plant-pollinator interactions between exotic and native plants
- Table S5. List of plant species and varieties encountered at all sites over the two years
- Table S6. List of pollinator species encountered at all sites over the two years

1. Supplementary Figures

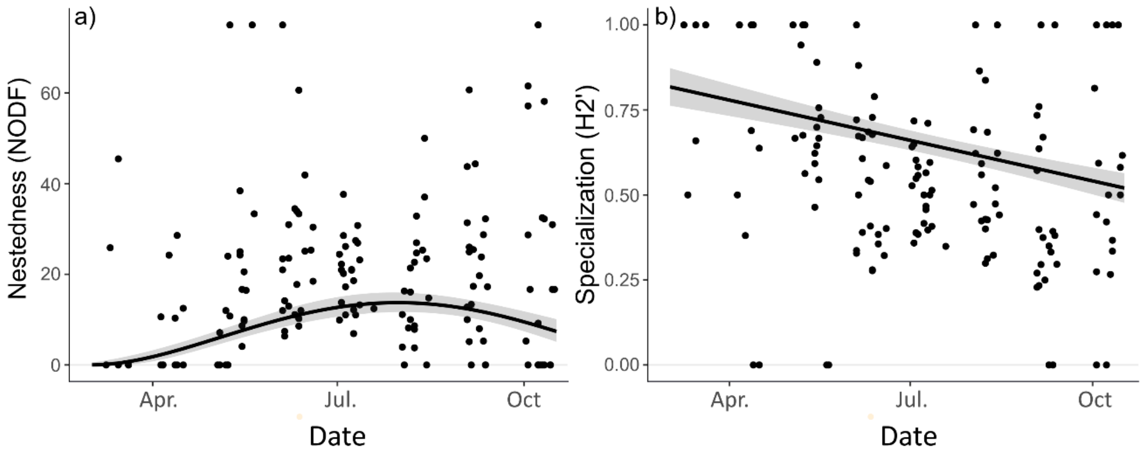

Figure S1. Seasonal variations in plant-pollinator interactions properties at network level: a) network-level nestedness (NODF), b) network-level index of interaction specialization H2'. The lines indicate predictions from the GLMM presented in Table S2 (±SE), points represent observed values. Predictions of both indices are modelled also accounting for the variations of flower density at community level. There was no significant effect of network size or green space size.

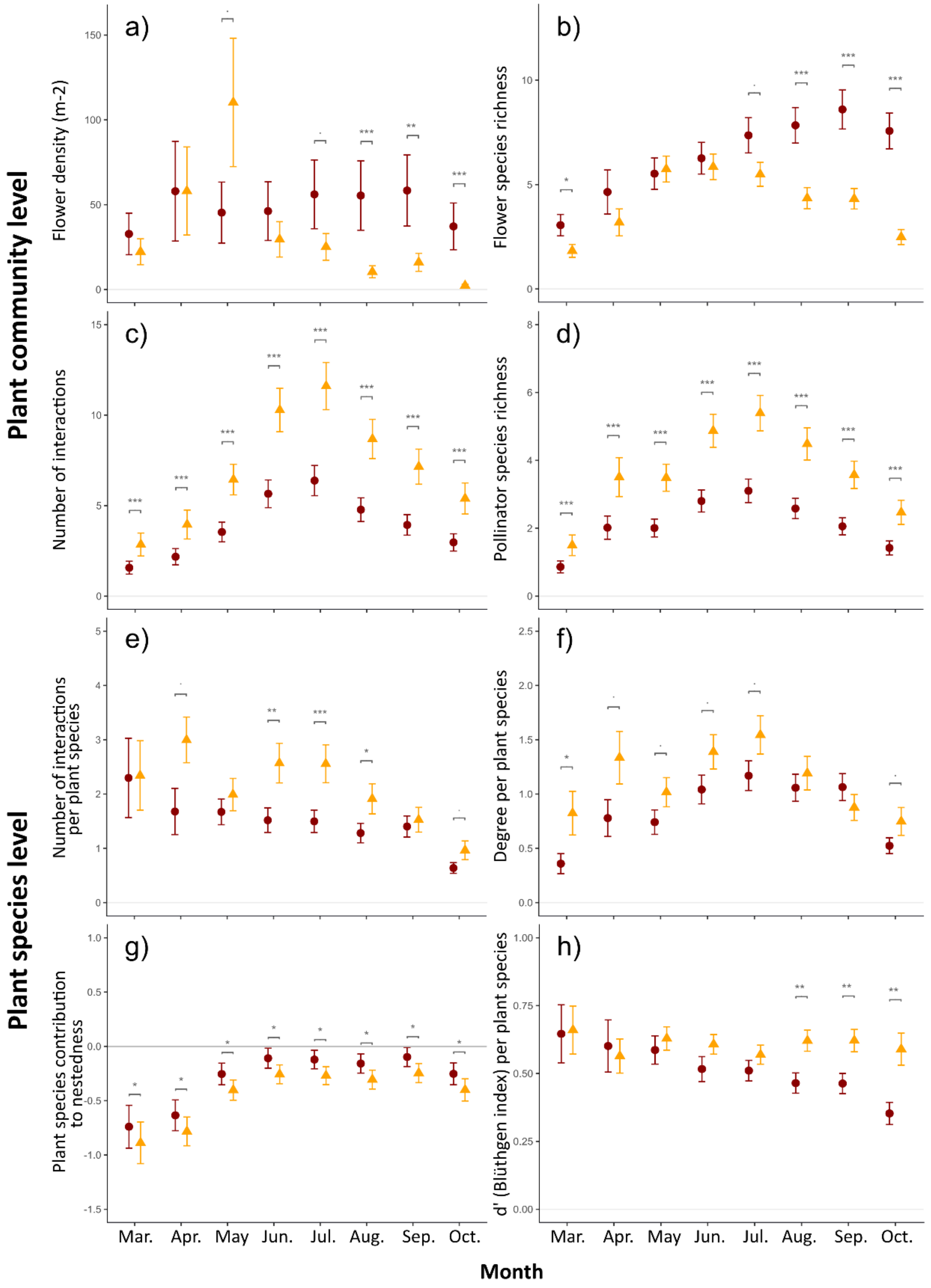

Figure S2. Seasonal variations in flower resource availability and plant-pollinator interactions, with time of year as a discrete variable, for exotic (red dots) and native (orange triangles) plants. Variables assessed at the plant community level : a) flower density per m² ; b) floral species richness per site ; c) number of plant-pollinator interactions ; d) number of pollinator species. Variables at the plant species level; e) number of interactions per plant species; f) number of interacting pollinator species per plant species (degree); g) contribution to monthly network nestedness per plant species; h) specialization index d' per plant species. Symbols represent estimated marginal means (±SE). Stars represent significant pairwise differences between exotic and native plants : · *P*<0.01 ; * *P*<0.05 ; ** *P*<0.01 ; *** *P*<0.001

2. Supplementary Tables

Table S1. Detailed information on the 12 selected green spaces and their flower resources. Plant species richness is aggregated over the two years of survey. Mean flower density is the average number of floral units per m² calculated per month.

Green space name	Coordinates	Green space size (m²)	Plant species richness (% native)	Mean flower density (/m²) (% native)
Jardin Abbé-Pierre	48.8289° 2.3803°	7,245	30 (93%)	31.2 (99%)
Parc de Belleville	48.8714° 2.385°	44,665	75 (31%)	43.8 (23%)
Parc de Bercy	48.8358° 2.3827°	58,786	56 (50%)	74.8 (64%)
Parc Georges Brassens	48.8315° 2.2996°	58,540	72 (36%)	40.0 (9%)
Parc André Citroen	48.8419° 2.2746°	50,126	58 (55%)	71.3 (8%)
Jardin des Plantes	48.844° 2.3614°	161,540	54 (67%)	90.3 (91%)
Jardin Catherine Labouré	48.8513° 2.3208°	21,715	54 (33%)	31.6 (10%)
Parc Monceau	48.8791° 2.3091°	97,361	65 (37%)	28.6 (35%)
Réservoir Montsouris	48.8247° 2.3326°	29,828	21 (95%)	8.1 (100%)
Jardin Pierre Emmanuel	48.8587° 2.3989°	61,547	39 (97%)	14.7 (98%)
Jardin Saint-Vincent	48.8882° 2.3413°	7,809	29 (97%)	7.5 (100%)
Jardin Villemin	48.875° 2.3611°	17,212	57 (25%)	44.4 (36%)

Table S2. GLMM of the network-level seasonal variations in plant-pollinator interaction nestedness (NODF) and specialization index (H2'). The predictors are given after variable selection. X^2 and associated *P*-values give the results of Type-II Wald analysis of deviance; df: degrees of freedom of the X^2 test. AICc: Second-order Akaike Information Criterion ; r^2 is the conditional r-squared value considering both the fixed and random effects.

Response variable and predictors	X^2	P	Estimates
**NESTEDNESS**			
(Quasi-Poisson, AICc=1141, r^2=0.34)			
Day^2	27.3 (2df)	<.0001	see Fig. S1
Flower density (log)	15.6 (1df)	.0001	slope: 0.43±0.11
Year	6.5 (1df)	.01	2.3±0.1 (2019), 2.7±0.1 (2020)
**SPECIALIZATION H2'**			
(Gaussian, AICc=3.5, r^2=0.18)			
Day	13.9 (1df)	.0002	see Fig. S1
Flower density (log)	11.5 (1df)	.0007	slope: -0.10±0.03
Year	10.9 (1df)	.001	0.7±0.03 (2019), 0.5±0.03 (2020)

Table S3. GLMM of seasonal variations in flower resource availability and plant-pollinator interactions, with time of year as a discrete variable (Month). Response variables are assessed at both community level and plant level, for native and exotic plant species (Origin). The predictors are given after variable selection. X^2 and associated *P*-values give the results of Type-III Wald analysis of deviance; df: degrees of freedom of the X^2 test. AICc: Second-order Akaike Information Criterion ; r^2 is the conditional r-squared value considering both the fixed and random effects.

Response variable and predictors	X^2	P	Estimates
	**Plant community level**		
**FLOWER DENSITY**			
(Negative binomial, AICc = 3921, r^2 = 0.40)			
Month	47.7 (7df)	<.0001	see Fig. S2
Origin	16.3 (1df)	<.0001	see Fig. S2
Month x Origin	40.5 (7df)	<.0001	see Fig. S2
Year	10.0 (1df)	.0015	5.1±0.3 (2019), 4.6±0.3 (2020)
**FLORAL SPECIES RICHNESS**			
(Negative binomial, AICc = 1802, r^2 = 0.29)			
Month	71.4 (7df)	<.0001	see Fig. S2
Origin	39.4 (1df)	<.0001	see Fig. S2
Month x Origin	32.8 (7df)	<.0001	see Fig. S2
**NUMBER OF INTERACTIONS**			
(Quasi-Poisson, AICc = 1969, r^2 = 0.77)			
Month	75.4 (7df)	<.0001	see Fig. S2
Origin	44.6 (1df)	<.0001	see Fig. S2
Flower density (log)	100.8 (1df)	<.0001	slope: 0.60±0.06
Floral species richness	133.0 (1df)	<.0001	slope: 0.54±0.05
Green space size	11.5 (1df)	.00071	slope: 0.17±0.05
**POLLINATOR RICHNESS**			
(Quasi-Poisson, AICc = 1463, r^2 = 0.71)			
Month	64.1 (7df)	<.0001	see Fig. S2
Origin	49.4 (1df)	<.0001	see Fig. S2
Flower density (log)	41.7 (1df)	<.0001	slope: 0.33±0.05
Floral species richness	154.6 (1df)	<.0001	slope: 0.51±0.04
Green space size	11.9 (1df)	.00057	slope: 0.14±0.04
	**Plant species level**		
**NUMBER OF INTERACTIONS**			
(Poisson, AICc = 6499, r^2 = 0.55)			
Month	122.4 (7df)	<.0001	see Fig. S2
Origin	5.1 (1df)	.024	see Fig. S2
Month x Origin	19.3 (7df)	.0073	see Fig. S2
Flower density /species (log)	310.1 (1df)	<.0001	slope: 0.37±0.02
Year	8.2 (1df)	.0041	0.6±0.2 (2019), 0.5±0.1 (2020)
**POLLINATOR RICHNESS (degree)**			
(Poisson, AICc = 4741, r^2 = 0.43)			
Month	77.6 (7df)	<.0001	see Fig. S2
Origin	7.8 (1df)	.0052	see Fig. S2
Month x Origin	15.9 (7df)	.026	see Fig. S2
Flower density /species (log)	64.2 (1df)	<.0001	slope: 0.21±0.03

**CONTRIBUTION TO NESTEDNESS**			
(Gaussian, AICc = 2224, r^2 = 0.19)			
Month	26.8 (7df)	.00037	see Fig. S2
Origin	4.4 (1df)	.035	see Fig. S2
Flower density /species (log)	4.1 (1df)	.042	slope: 0.05±0.03
Year	7.3 (1df)	.0071	-0.4±0.1 (2019), -0.3±0.2 (2020)
**SPECIALIZATION (d')**			
(Beta-regression, AICc = -1658, r^2 = 0.69)			
Month	10.4 (7df)	.17	see Fig. S2
Origin	7.2 (1df)	.0073	see Fig. S2
Month x Origin	8.9 (7df)	.26 (NS)	see Fig. S2
Year	5.9 (1df)	.015	0.4±0.1 (2019), 0.1±0.1 (2020)

Table S4. Post-hoc comparisons of flower resource availability and plant-pollinator interactions between exotic and native plants (pairwise comparisons per month). Values shown are the exotic/native ratio (±SE) for each response variable, with the *P*-value of the pairwise test with "fdr" adjustment (in brackets). Red boxes indicate that the value of the response variable is significantly higher for exotic plants, while orange boxes indicate that the value is significantly higher for native plants.

**Response variable**	**Mar.**	**Apr.**	**May**	**Jun.**	**Jul.**	**Aug.**	**Sep.**	**Oct.**
**Plant community level : ratio exotic / native**								
Flower density df=364	1.47±0.68 (0.40)	1.00±0.63 (1.00)	0.41±0.21 (0.071)	1.56±0.79 (0.38)	2.23±0.98 (0.067)	5.32±2.39 (0.0002)	3.64±1.58 (0.0032)	16.43±7.40 (<.0001)
Floral species richness df=365	1.67±0.39 (0.028)	1.46±0.44 (0.21)	0.96±0.16 (0.82)	1.07±0.17 (0.66)	1.34±0.20 (0.052)	1.80±0.28 (0.0002)	1.99±0.30 (<.0001)	3.04±0.55 (<.0001)
Number of interactions df=369	0.56±0.05 (<.0001)	0.56±0.05 (<.0001)	0.56±0.05 (<.0001)	0.56±0.05 (<.0001)	0.56±0.05 (<.0001)	0.56±0.05 (<.0001)	0.56±0.05 (<.0001)	0.56±0.05 (<.0001)
Pollinator richness df=341	0.59±0.04 (<.0001)	0.59±0.04 (<.0001)	0.59±0.04 (<.0001)	0.59±0.04 (<.0001)	0.59±0.04 (<.0001)	0.59±0.04 (<.0001)	0.59±0.04 (<.0001)	0.59±0.04 (<.0001)
**Plant species level : ratio exotic / native**								
Number of interactions df=1573	0.98±0.41 (0.96)	0.56±0.17 (0.051)	0.84±0.15 (0.34)	0.59±0.10 (0.0022)	0.59±0.09 (0.0010)	0.67±0.11 (0.019)	0.92±0.16 (0.63)	0.66±0.14 (0.051)
Pollinator richness (degree) df=1574	0.44±0.15 (0.017)	0.59±0.16 (0.054)	0.73±0.14 (0.095)	0.75±0.11 (0.060)	0.76±0.11 (0.054)	0.89±0.14 (0.45)	1.21±0.20 (0.23)	0.70±0.15 (0.086)
Contribution to nestedness df=954	0.15±0.07 (0.035)	0.15±0.07 (0.035)	0.15±0.07 (0.035)	0.15±0.07 (0.035)	0.15±0.07 (0.035)	0.15±0.07 (0.035)	0.15±0.07 (0.035)	0.15±0.07 (0.035)
Specialization (d') df=932	0.94±0.57 (0.92)	1.17±0.55 (0.74)	0.84±0.23 (0.52)	0.69±0.16 (0.12)	0.79±0.16 (0.25)	0.53±0.12 (0.0040)	0.53±0.12 (0.0048)	0.38±0.11 (0.0013)

Table S5. List of plant species and varieties encountered at all sites and during the two years of monitoring. For each one is given its origin (E: exotic, N: native ; arch.: archaeophyte, hort.: horticultural variety, nat.: naturalized, inv.: invasive), its management type (P: planted, S: spontaneous, SubS: subspontaneous), its growth form (Pe: perennial, An: annual, Bi: biennial), the total number of pollinator visits, the total number of floral units and the number of interacting wild (non-domesticated) pollinator taxa. Plant species and varieties with no count of floral units were present in the survey transects but not in the survey quadrats.

Plant species or variety	Origin	Management	Growth form	Total number of floral units	Total number of visits	Wild pollinator taxa
Acanthus mollis	E	P	Pe	523	13	4
Achillea filipendulina	E	SubS	Pe	14	6	4
Achillea millefolium	N	S	Pe	80	22	17
Achillea ptarmica	N	P	Pe		2	1
Acmella oleracea	E	P	Pe	512	9	3
Aesculus parviflora	E	P	Pe	10	20	6
Agastache foeniculum	E	P	Pe		3	2
Agastache mexicana	E	P	Pe	111	6	2
Ageratum houstonianium	E	P	An	160	0	0
Ajuga reptans	N	S	Pe		1	1
Alliara petiolata	N	S	Bi	53	0	0
Allium ampeloprasum	E	P	Pe	1	4	3
Allium nigrum	E	P	Pe	115	6	0
Allium sphaerocephalon	N	P	Pe		4	3
Althaea officinalis	N (arch.)	S	Pe	35	7	1
Amaranthus cruentus	E	SubS	An	165	1	1
Amaryllis belladona	E	P	Pe	13	0	0
Anacamptis pyramidalis	N	S	Pe	8	0	0
Anaphalis margaritacea	E	P	Pe		3	3
Anemone blanda	E	SubS	Pe	30	0	0
Anemone hupehensis	E	P	Pe		3	2
Anemone nemorosa	N	S	Pe	1	1	1
Anemone x blanda	E (hort.)	P	Pe	2	0	0
Angelica archangelica	E	P	Bi		1	1
Anthemis cotula	N	P	An	6	0	0
Anthericum liliago	N	P	Pe	5	1	1
Anthriscus sylvestris	N	S	An	368	20	9
Antirrhinum majus	E	P	Pe		2	1
Aquilegia alpina	E	P	Pe	16	3	1
Aquilegia vulgaris	N	P	Pe	8	0	0
Arabis caucasica	E	P	Pe	41	0	0
Arctium lappa	N	S	Bi	2	2	1
Artemisia absinthium	N (arch.)	S	Pe		2	0
Artemisia annua	E (nat.)	S	An		1	1
Aruncus dioicus	E	P	Pe		11	6
Asparagus aethiopicus	E	P	Pe		2	1
Ballota nigra	N	S	Pe	52	4	1
Begonia cucullata	E	P	Pe	30	1	0
Begonia x samperflorens	E	P	Pe	136	0	0
Bellis perennis	N	S	Pe	845	46	24
Bellis perennis (double)	E (hort.)	P	Pe	28	1	0
Berberis aquifolium	E (inv.)	P	Pe	7	0	0
Bidens ferulifolia	E	P	An	281	15	7
Bidens pilosa	E	P	An	100	10	4
Borago officinalis	E	SubS	An	86	64	9
Brunnera macrophylla	E	P	Pe	69	0	0
Bryonia dioica	N	S	Pe	15	15	2
Buddleja davidii	E (inv.)	P	Pe	978	11	2
Buxus sempervirens	N (arch.)	S	Pe		5	0
Calceolaria integrifolia	E	P	Pe	35	6	4
Calendula officinalis	E	P	Pe	17	2	1
Calluna vulgaris	N	P	Pe	2,959	4	2
Campanula medium	E	P	Bi	9	0	0
Campanula muralis	E	P	Pe		2	1
Campanula persicifolia	N	P	Pe	1	7	3
Campanula poscharskyana	E	P	Pe	147	9	2
Campanula pyramidalis	E	P	Pe		24	4
Campanula rapunculoides	N (arch.)	S	Pe	12	5	4
Canna indica	E	P	Pe	25	1	1
Cardamine hirsuta	N	S	An	10	0	0
Catananche caerulea	E	SubS	Pe	90	12	5

Centaurea nigra	N	S	Pe		13	5
Centranthus ruber	N (arch.)	SubS	Pe	112	0	0
Cerinthe major	E	P	An		2	1
Chaerophyllum temulum	N	S	Bi	34	0	0
Chelidonium majus	N	S	Pe		1	1
Chionodoxa forbesii	E	P	Pe	2	1	0
Cichorium intybus	N	S	Pe		6	4
Cirsium arvense	N	S	Pe	6	17	11
Cirsium vulgare	N	S	Pe		1	1
Clematis vitalba	N	S	Pe		5	3
Clematis viticella	E	P	Pe	7	0	0
Colchicum autumnale	N	P	Pe	1	0	0
Convolvulus arvensis	N	S	Pe	22	14	5
Convolvulus tricolor	E	P	An	18	1	1
Coreopsis grandiflora	E	P	Pe		8	3
Coreopsis tinctoria	E	P	An	33	2	1
Cornus florida	E	P	Pe	17	0	0
Cornus kousa	E	P	Pe	10	0	0
Cornus sanguinea	N	P	Pe	10,425	91	8
Coronilla varia	N	S	Pe	1,036	41	7
Cosmos bipinnatus	E	P	An	41	11	6
Cosmos sulphureus	E	P	An	11	1	1
Cota tinctoria	E (nat.)	SubS	Bi		2	1
Cotoneaster coriaceus	E	P	Pe	136	3	1
Crataegus monogyna	N	P	Pe	287	9	4
Crepis biennis	N	S	Bi	1	13	7
Crepis capillaris	N	S	An	25	6	4
Crocosmia crocosmiiflora	E	P	Pe	162	8	2
Cuphea ignea	E	P	Pe	94	2	2
Cyanus segetum	N	S	An		1	1
Cyclamen hederifolium	E	SubS	Pe	18	1	1
Cymbalaria muralis	N	S	Pe		3	2
Cynara cardunculus	E	P	Pe		9	5
Cynara scolymus	E	P	Pe		6	2
Cytisus villosus	E	P	Pe		3	1
Dahlia pinnata	E	P	Pe	8	11	1
Dahlia pinnata (double)	E	P	Pe		1	1
Daucus carota	N	S	Bi	31	52	21
Delphinium elatum	E	P	Pe	6	0	0
Dianthus barbatus	E	P	Pe	592	0	0
Dianthus carthusianorum	N	S	Pe	281	52	16
Digitalis purpurea	N	P	Bi		5	3
Dipsacus fullonum	N	S	Bi	4	15	8
Duchesnea indica	E (nat.)	S	Pe		2	2
Echinops sphaerocephalus	E	P	Pe		17	7
Echium vulgare	N	S	Bi	39	5	2
Emilia coccinea	E	P	Pe		2	0
Epilobium angustifolium	N	P	Pe	2	1	0
Epilobium ciliatum	E (nat.)	S	Pe	4	0	0
Epilobium hirsutum	N	S	Pe	11	6	3
Erica cinerea	N	P	Pe	2,425	9	2
Erigeron annuus	E (inv.)	S	An	4	0	0
Erigeron canadensis	E (inv.)	S	An	42	27	10
Erigeron karvinskianus	E	P	Pe	2,129	85	21
Erinus alpinus	E	P	Pe	3	0	0
Erodium cicutarium	N	S	Bi	28	0	0
Erysimum cheiri	N (arch.)	SubS	Pe	1,444	12	6
Eschscholzia californica	E	P	An	1	3	3
Euonymus japonicus	E	P	Pe	615	20	12
Euphorbia amygdaloides	N	P	Pe		7	6
Euphorbia characias	E	P	Pe	3,213	78	14
Euphorbia hypericifolia	E	P	Pe	1,946	21	11
Euphorbia rotundifolia	E	P	Pe		5	3
Exochorda racemosa	E	P	Pe	20	1	0
Fallopia convolvulus	N	S	An	223	0	0
Felicia amelloides	E	P	Pe	1,194	15	11
Ficaria ranunculoides	N	S	Pe	1	1	1
Foeniculum vulgare	N (arch.)	SubS	Pe	104	16	7
Fuchsia magellanica	E	P	Pe	1,405	38	7
Gaillardia aristata	E	P	An	68	27	2
Gaillardia pulchella	E	P	An	22	2	2
Galega officinalis	E (inv.)	S	Pe	34	4	2
Galium aparine	N	S	An	10	0	0
Galium verum	N	S	Pe		1	1
Gazania rigens	E	P	Pe	2	1	1
Genista tinctoria	N	P	Pe		2	1

Geranium dissectum	N	S	An	39	0	0
Geranium endressii	E	P	Pe	210	10	8
Geranium molle	N	S	An	25	1	0
Geranium phaeum	E	P	Pe	10	4	0
Geranium robertianum	N	S	Bi	140	14	5
Geranium rotundifolium	N	S	An	1	2	1
Geranium sanguineum	N	P	Pe	53	11	8
Geum coccineum	E	P	Pe	5	0	0
Geum urbanum	N	S	Pe	13	0	0
Glebionis segetum	N	S	An		4	3
Glechoma hederacea	N	S	Pe	1,385	24	7
Gomphrena globosa	E	P	An	27	2	2
Hebe speciosa	E	P	Pe		55	10
Hedera helix	N	S	Pe	8	10	6
Helianthemum apenninum	N	S	Pe		3	2
Helianthemum nummularium	N	P	Pe	2	2	1
Helleborus foetidus	N	P	Pe	8	1	0
Helleborus x ericsmithii	E	P	Pe	94	0	0
Helminthotheca echioides	N	S	An	168	171	21
Heracleum sphondylium	N	S	Pe	9	7	6
Hesperis matronalis	N (arch.)	S	Bi	41	2	2
Heuchera sanguinea	E	P	Pe	692	0	0
Hibiscus syriacus	E	P	Pe		23	5
Hibiscus trionum	E	P	An	5	1	0
Himantoglossum hircinum	N	S	Pe	38	0	0
Honorius nutans	E	SubS	Pe	1	0	0
Hosta plantaginea	E	P	Pe	14	2	2
Hyacinthus orientalis	E	P	Pe	37	0	0
Hydrangea paniculata	E	P	Pe	34	2	0
Hypericum androsaemum	N	P	Pe		11	2
Hypericum perforatum	N	S	Pe	18	22	13
Hypochaeris radicata	N	S	Pe	69	55	16
Iberis sempervirens	E	P	Pe	18	3	0
Impatiens balfourii	E (inv.)	P	An	291	28	6
Impatiens walleriana	E	P	Pe	21	0	0
Indigofera tinctoria	E	P	Pe		17	4
Inula helenium	N (arch.)	S	Pe		5	3
Ipomoea cairica	E	P	Pe	2	1	1
Iris germanica	E	P	Pe	2	0	0
Isotoma axillaris	E	P	Pe	440	5	2
Jacobaea maritima	E	P	Pe	222	29	8
Jasminum nudiflorum	E	P	Pe		14	2
Kniphofia uvaria	E	P	Pe	300	24	3
Koelreuteria paniculata	E	P	Pe		7	4
Lactuca serriola	N	S	An	4	0	0
Lamium album	N	S	Pe	33	9	2
Lamium purpureum	N	S	An	23	5	4
Lantana camara	E	P	Pe	89	5	2
Lapsana communis	N	S	An	8	11	7
Lathyrus latifolius	N (arch.)	S	Pe	23	27	7
Lepidium draba	N (arch.)	S	Pe		1	1
Levisticum officinale	E	SubS	Pe		2	2
Leycesteria formosa	E	P	Pe	59	9	1
Ligustrum vulgare	N	P	Pe	170	12	2
Linaria maroccana	E	P	An	30	4	2
Linaria vulgaris	N	S	Pe	55	0	0
Lobelia cardinalis	E	P	Pe	14	2	1
Lobelia erinus	E	P	Pe	3	0	0
Lobelia incana	E	P	Pe	48	3	1
Lobelia siphilitica	E	P	Pe	83	2	2
Lobularia maritima	E	SubS	An	519	2	1
Lonicera caprifolium	E (nat.)	SubS	Pe		12	4
Lonicera periclymenum	N	S	Pe		2	2
Lonicera xylosteum	N	P	Pe		1	0
Lotus corniculatus	N	S	Pe	254	47	11
Lysimachia arvensis	N	S	An	2	0	0
Lysimachia foemina	N	P	An	13	2	2
Lysimachia thyrsiflora	E	P	Pe	2	0	0
Lythrum salicaria	N	P	Pe		1	1
Malcolmia maritima	E	P	An		2	2
Malus sp	N	P	Pe		3	0
Malva alcea	N	P	Pe		2	1
Malva sylvestris	N	S	Bi	8	38	14
Matthiola incana	E	P	An	53	0	0
Matthiola incana (double)	E (hort.)	P	An	28	0	0
Medicago arabica	N	S	An	3	0	0

Medicago lupulina	N (arch.)	S	Bi	100	0	0
Medicago sativa	N (arch.)	S	Pe	206	59	13
Melilotus officinalis	N	S	Bi		15	6
Melissa officinalis	E	P	Pe		2	2
Mentha suaveolens	N	P	Pe	708	17	10
Mirabilis jalapa	E	SubS	Pe	9	1	1
Muscari neglectum	N	S	Pe	121	2	2
Myosotis arvensis	N	S	An	21	0	0
Myosotis sylvatica	E	P	Bi	3,705	12	6
Narcissus pseudonarcissus	N	P	Pe	33	4	3
Narcissus pseudonarcissus (double)	E (hort.)	P	Pe	9	0	0
Nepeta cataria	N	P	Pe		2	1
Nicotiana tabacum	E	P	An	199	6	2
Nicotiana x alata	E	P	An	6	0	0
Nigella damascena	E	P	An	22	4	1
Oenothera biennis	E (nat.)	S	Bi	2	4	0
Oenothera glazioviana	E (nat.)	S	Bi	2	18	7
Oenothera lindheimeri	E	P	Pe	305	68	23
Origanum vulgare	N	P	Pe	472	27	9
Orlaya grandifolia	N	P	An	1	0	0
Orobanche hederae	N	S	Pe	101	4	3
Osteospermum ecklonis	E	P	Pe	47	4	3
Oxalis corniculata	N (arch.)	S	Pe	9	4	4
Paeonia officinalis	E	P	Pe		5	0
Papaver nudicaule	E	P	Bi	17	4	2
Papaver rhoeas	N	S	An	4	3	2
Pelargonium inquinans	E	P	Pe	479	11	2
Pelargonium x hortorum	E	P	Pe	45	0	0
Pentas lanceolata	E	P	Pe	130	4	2
Perilla frutescens	E	P	Pe		1	1
Perovskia atriplicifolia	E	P	Pe	535	47	6
Persicaria orientalis	E	P	Pe	24	1	0
Petunia axillaris	E	P	An	26	0	0
Phacelia tanacetifolia	E	SubS	An	61	97	11
Philadelphus coronarius	E	P	Pe	5	1	1
Phlomis herba venti	E	P	An	18	0	0
Phuopsis stylosa	E	P	Pe	17	3	3
Physostegia virginiana	E	P	Pe	6	0	0
Phytolacca acinosa	E	P	Pe	28	0	0
Picris hieracioides	N	S	Bi	98	65	20
Pimpinella saxifraga	N	S	Pe		11	6
Plantago lanceolata	N	S	Pe	279	10	1
Plantago major	N	S	Pe	11	2	1
Potentilla reptans	N	S	Pe	94	50	19
Primula veris	N	S	Pe	94	1	0
Primula vulgaris	E (hort.)	P	Pe	572	3	3
Primula vulgaris (double)	E (hort.)	P	Pe	18	0	0
Prunella vulgaris	N	S	Pe	167	2	1
Prunus spinosa	N	P	Pe	558	12	5
Pulmonaria officinalis	E	P	Pe	33	3	2
Ranunculus acris	N	S	Pe	22	4	2
Ranunculus bulbosus	N	S	Pe	119	28	13
Ranunculus repens	N	S	Pe	215	31	17
Reseda lutea	N	S	Pe		20	3
Rosa canina	N	P	Pe	4	9	3
Rubus fruticosus	N	P	Pe	11	27	8
Rubus occidentalis	E	P	Pe	15	4	2
Rudbeckia fulgida	E	P	Pe	4	1	1
Rudbeckia hirta	E	P	Pe	52	27	12
Rumex acetosella	N	S	Pe		1	0
Salvia coccinea	E	P	Pe	246	28	6
Salvia farinacea	E	P	Pe	338	27	7
Salvia involucrata	E	P	Pe	27	0	0
Salvia microphylla	E	P	Pe	255	84	10
Salvia patens	E	P	Pe	42	25	3
Salvia pratensis	N	S	Pe	18	8	4
Salvia splendens	E	P	Pe	163	0	0
Salvia uliginosa	E	P	Pe	189	62	11
Salvia viridis	E	P	An	33	1	1
Sambucus nigra	N	P	Pe	541	2	1
Sanguisorba minor	N	S	Pe	12	0	0
Saponaria officinalis	N	S	Pe	8	1	0
Saxifraga x arendsii	E	P	Pe	254	1	1
Scabiosa atropurpurea	E	P	Pe	9	0	0
Scabiosa ochroleuca	E	P	Pe		4	3
Sedum rupestre	N	P	Pe	38	3	3

Senecio inaequidens	E (inv.)	S	Pe		4	2
Senecio jacobaea	N	S	An	185	47	24
Seseli montanum	N	S	Bi		1	1
Silene coronaria	E	P	Pe	47	2	2
Sinapis arvensis	N	S	An	4	0	0
Sisymbrium officinale	N	S	An	29	12	5
Solanum dulcamara	N	S	Pe	28	20	2
Solanum nigrum	N	S	Pe	1	0	0
Solanum pimpinellifolium	E	P	An	5	0	0
Solanum sisymbriifolium	E	P	An	22	16	5
Sonchus asper	N	S	An	9	5	3
Sonchus oleraceus	N	S	An	33	12	5
Sorbus latifolia	N	P	Pe		1	1
Spergularia rubra	N	S	An	2	0	0
Stachys byzantina	E	P	Pe	692	14	7
Stachys recta	N	S	Pe		37	5
Sternbergia lutea	E	P	Pe	2	0	0
Symphyotrichum novi-belgi	E	P	Pe	6	0	0
Tagetes erecta	E	P	An	23	2	1
Tagetes patula	E	P	An	87	4	2
Tanacetum vulgare	N	S	Pe		4	2
Taraxacum officinale	N	S	Pe	29	20	14
Thalictrum aquilegiifolium	E	P	Pe	2	4	1
Thymus serpyllum	N	P	Pe	1,361	96	9
Tithonia rotundifolia	E	P	Pe		7	4
Torilis arvensis	N	S	An	227	9	7
Torilis nodosa	N (arch.)	S	An	330	6	4
Trifolium dubium	N	S	An	7	0	0
Trifolium pratense	N (arch.)	S	Pe	3	2	1
Trifolium repens	N (arch.)	S	Pe	879	116	8
Tristagma uniflorum	E	P	Pe	39	0	0
Tropaeolum majus	E	P	An	13	0	0
Tulipa × gesneriana	E	P	Pe	8	3	2
Tulipa × gesneriana (double)	E (hort.)	P	Pe	1	0	0
Urospermum dalechampii	E	SubS	Pe		1	0
Urticia dioica	N	S	Pe	12	0	0
Verbascum blattaria	N	P	Bi	9	8	4
Verbascum thapsus	N	P	Bi	27	0	0
Verbena bonariensis	E	P	Pe	5,087	116	15
Verbena officinalis	N	S	Pe		1	1
Verbena rigida	E	P	Pe	869	8	3
Verbena tenera	E	P	Pe	656	6	1
Veronica chamaedrys	N	S	Pe		1	1
Veronica hederifolia	N	S	An	10	0	0
Veronica persica	N (arch.)	S	An	421	20	12
Veronicastrum virginicum	E	P	Pe	130	27	13
Viburnum lantana	N	P	Pe	2,054	3	1
Viburnum opulus	N	P	Pe	1,680	2	1
Viburnum tinus	E	P	Pe	15	0	0
Vicia sativa	N	S	An	8	0	0
Vicia sepium	N	S	Pe	33	0	0
Vinca minor	N	S	Pe	3	0	0
Viola x cornuta	E (hort.)	P	Pe	1,289	12	4
Viola x wittrockiana	E (hort.)	P	Pe	719	9	8
Visnaga daucoides	E	SubS	An	6	16	7
Zinnia angustifolia	E	P	An	15	4	2
Zinnia elegans	E	P	An	6	0	0

Table S6. List of insect pollinator species encountered at all sites and during the two years of monitoring. For each one is given its origin (E: exotic, N: native), the total number of recorded interactions with plants, the total number of plant species visited.

Species	Order	Family	Origin	Number of interactions	Number of visited plants
Coccinella septempunctata	Coleoptera	Coccinellidae	N	1	1
Oedemera nobilis	Coleoptera	Oedemeridae	N	9	4
Cetonia aurata	Coleoptera	Scarabaeidae	N	3	3
Trichius fasciatus	Coleoptera	Scarabaeidae	N	1	1
Bombylius discolor	Diptera	Bombyliidae	N	2	2
Bombylius major	Diptera	Bombyliidae	N	9	7
Villa hottentotta	Diptera	Bombyliidae	N	5	5
Cheilosa sp.	Diptera	Syrphidae	N	1	1
Epistrophe eligans	Diptera	Syrphidae	N	3	3
Episyrphus balteatus	Diptera	Syrphidae	N	78	37
Eristalis arbustorum	Diptera	Syrphidae	N	3	2
Eristalis similis	Diptera	Syrphidae	N	3	3
Eristalis tenax	Diptera	Syrphidae	N	9	7
Eumerus amoenus	Diptera	Syrphidae	N	1	1
Eupeodes corollae	Diptera	Syrphidae	N	10	9
Eupeodes luniger	Diptera	Syrphidae	N	23	18
Eupeodes sp.	Diptera	Syrphidae	N	1	1
Helophilus pendulus	Diptera	Syrphidae	N	2	2
Melanostoma mellinum	Diptera	Syrphidae	N	3	3
Melanostoma scalare	Diptera	Syrphidae	N	1	1
Meliscaeva auricollis	Diptera	Syrphidae	N	5	3
Merodon equestris	Diptera	Syrphidae	N	3	3
Myathropa florea	Diptera	Syrphidae	N	12	12
Paragus haemorrhous	Diptera	Syrphidae	N	1	1
Paragus pecchiolii	Diptera	Syrphidae	N	1	1
Pipiza festiva	Diptera	Syrphidae	N	1	1
Pipizella sp.	Diptera	Syrphidae	N	1	1
Platycheirus scutatus	Diptera	Syrphidae	N	14	13
Platycheirus sticticus	Diptera	Syrphidae	N	1	1
Scaeva pyrastri	Diptera	Syrphidae	N	2	2
Sphaerophoria rueppelli	Diptera	Syrphidae	N	1	1
Sphaerophoria scripta	Diptera	Syrphidae	N	64	32
Syritta pipiens	Diptera	Syrphidae	N	66	29
Syrphus rectus	Diptera	Syrphidae	N	1	1
Syrphus ribesii	Diptera	Syrphidae	N	5	5
Syrphus vitripennis	Diptera	Syrphidae	N	3	3
Volucella zonaria	Diptera	Syrphidae	N	2	2
Anthomyia sp.	Diptera	Anthomyiidae	N	1	1
Bibio sp.	Diptera	Bibionidae	N	2	2
Calliphora sp.	Diptera	Calliphoridae	N	56	3
Lucilia sp.	Diptera	Calliphoridae	N	8	5
Pollenia sp.	Diptera	Calliphoridae	N	1	1
Myopa sp.	Diptera	Conopidae	N	1	1

Physocephala sp.	Diptera	Conopidae	N	1	1
Sicus ferrugineus	Diptera	Conopidae	N	1	1
Fannia sp.	Diptera	Fanniidae	N	5	3
Suillia sp.	Diptera	Heleomyzidae	N	1	1
Graphomya sp.	Diptera	Muscidae	N	2	2
Musca sp.	Diptera	Muscidae	N	1	1
Phaonia sp.	Diptera	Muscidae	N	3	1
Polietes sp.	Diptera	Muscidae	N	1	1
Stevenia sp.	Diptera	Rhinophoridae	N	1	1
Sarcophaga sp.	Diptera	Sarcophagidae	N	6	3
Scathophaga sp.	Diptera	Scathophagidae	N	11	2
Scathophaga stercoraria	Diptera	Scathophagidae	N	1	1
Cylindromyia sp.	Diptera	Tachinidae	N	3	1
Phasia sp.	Diptera	Tachinidae	N	2	1
Tachina fera	Diptera	Tachinidae	N	4	3
Tachina sp.	Diptera	Tachinidae	N	2	2
Andrena bicolor	Hymenoptera	Andrenidae	N	7	7
Andrena dorsata	Hymenoptera	Andrenidae	N	9	7
Andrena flavipes	Hymenoptera	Andrenidae	N	14	10
Andrena florea	Hymenoptera	Andrenidae	N	9	1
Andrena gravida	Hymenoptera	Andrenidae	N	8	6
Andrena minutula	Hymenoptera	Andrenidae	N	23	19
Andrena minutuloides	Hymenoptera	Andrenidae	N	6	4
Andrena nitida	Hymenoptera	Andrenidae	N	4	4
Andrena propinqua	Hymenoptera	Andrenidae	N	1	1
Andrena proxima	Hymenoptera	Andrenidae	N	5	2
Andrena pusilla	Hymenoptera	Andrenidae	N	4	4
Andrena tenuistriata	Hymenoptera	Andrenidae	N	1	1
Andrena ventralis	Hymenoptera	Andrenidae	N	1	1
Andrena viridescens	Hymenoptera	Andrenidae	N	1	1
Panurgus dentipes	Hymenoptera	Andrenidae	N	2	1
Anthophora plumipes	Hymenoptera	Apidae	N	53	19
Anthophora quadrimaculata	Hymenoptera	Apidae	N	8	5
Apis mellifera	Hymenoptera	Apidae	N	1107	153
Bombus hortorum	Hymenoptera	Apidae	N	2	2
Bombus hypnorum	Hymenoptera	Apidae	N	3	3
Bombus lapidarius	Hymenoptera	Apidae	N	18	15
Bombus lucorum	Hymenoptera	Apidae	N	36	16
Bombus pascuorum	Hymenoptera	Apidae	N	525	116
Bombus pratorum	Hymenoptera	Apidae	N	9	6
Bombus terrestris	Hymenoptera	Apidae	N	94	41
Bombus vestalis	Hymenoptera	Apidae	N	15	6
Nomada flava	Hymenoptera	Apidae	N	1	1
Nomada flavoguttata	Hymenoptera	Apidae	N	14	8
Xylocopa violacea	Hymenoptera	Apidae	N	24	11
Colletes daviesanus	Hymenoptera	Colletidae	N	2	2
Colletes hederae	Hymenoptera	Colletidae	N	1	1
Colletes marginatus	Hymenoptera	Colletidae	N	1	1

Colletes similis	Hymenoptera	Colletidae	N	3	2
Hylaeus communis	Hymenoptera	Colletidae	N	46	28
Hylaeus gredleri	Hymenoptera	Colletidae	N	2	2
Hylaeus hyalinatus	Hymenoptera	Colletidae	N	1	1
Hylaeus incongruus	Hymenoptera	Colletidae	N	1	1
Hylaeus pictipes	Hymenoptera	Colletidae	N	1	1
Hylaeus punctatus	Hymenoptera	Colletidae	N	44	13
Hylaeus signatus	Hymenoptera	Colletidae	N	18	1
Hylaeus variegatus	Hymenoptera	Colletidae	N	1	1
Halictus langobardicus	Hymenoptera	Halictidae	N	2	2
Halictus maculatus	Hymenoptera	Halictidae	N	1	1
Halictus scabiosae	Hymenoptera	Halictidae	N	39	18
Halictus simplex	Hymenoptera	Halictidae	N	8	7
Halictus subauratus	Hymenoptera	Halictidae	N	17	10
Halictus tumulorum	Hymenoptera	Halictidae	N	29	19
Lasioglossum bluethgeni	Hymenoptera	Halictidae	N	2	2
Lasioglossum calceatum	Hymenoptera	Halictidae	N	20	12
Lasioglossum fulvicorne	Hymenoptera	Halictidae	N	1	1
Lasioglossum glabriusculum	Hymenoptera	Halictidae	N	36	8
Lasioglossum griseolum	Hymenoptera	Halictidae	N	1	1
Lasioglossum laticeps	Hymenoptera	Halictidae	N	49	27
Lasioglossum leucozonium	Hymenoptera	Halictidae	N	30	11
Lasioglossum limbellum	Hymenoptera	Halictidae	N	5	4
Lasioglossum malachurum	Hymenoptera	Halictidae	N	12	10
Lasioglossum minutissimum	Hymenoptera	Halictidae	N	7	6
Lasioglossum morio	Hymenoptera	Halictidae	N	74	34
Lasioglossum nitidulum	Hymenoptera	Halictidae	N	18	11
Lasioglossum pallens	Hymenoptera	Halictidae	N	1	1
Lasioglossum pauxillum	Hymenoptera	Halictidae	N	39	19
Lasioglossum politum	Hymenoptera	Halictidae	N	26	18
Lasioglossum pygmaeum	Hymenoptera	Halictidae	N	2	2
Lasioglossum villosulum	Hymenoptera	Halictidae	N	146	17
Sphecodes crassus	Hymenoptera	Halictidae	N	1	1
Sphecodes ephippius	Hymenoptera	Halictidae	N	1	1
Sphecodes ferruginatus	Hymenoptera	Halictidae	N	1	1
Sphecodes gibbus	Hymenoptera	Halictidae	N	1	1
Sphecodes pseudofasciatus	Hymenoptera	Halictidae	N	1	1
Sphecodes puncticeps	Hymenoptera	Halictidae	N	1	1
Anthidiellum strigatum	Hymenoptera	Megachilidae	N	2	2
Anthidium manicatum	Hymenoptera	Megachilidae	N	43	8
Anthidium septemspinosum	Hymenoptera	Megachilidae	N	53	23
Chelostoma campanularum	Hymenoptera	Megachilidae	N	1	1
Chelostoma rapunculi	Hymenoptera	Megachilidae	N	2	2
Coelioxys inermis	Hymenoptera	Megachilidae	N	2	2
Heriades crenulata	Hymenoptera	Megachilidae	N	2	2
Heriades truncorum	Hymenoptera	Megachilidae	N	14	10
Megachile centuncularis	Hymenoptera	Megachilidae	N	63	43
Megachile ericetorum	Hymenoptera	Megachilidae	N	2	1

Megachile lagopoda	Hymenoptera	Megachilidae	N	8	5
Megachile maritima	Hymenoptera	Megachilidae	N	2	2
Megachile rotundata	Hymenoptera	Megachilidae	N	9	5
Megachile willughbiella	Hymenoptera	Megachilidae	N	95	27
Osmia bicornis	Hymenoptera	Megachilidae	N	14	12
Osmia caerulescens	Hymenoptera	Megachilidae	N	3	2
Osmia cornuta	Hymenoptera	Megachilidae	N	3	2
Pseudoanthidium nanum	Hymenoptera	Megachilidae	N	1	1
Stelis punctulatissima	Hymenoptera	Megachilidae	N	5	4
Macropis europaea	Hymenoptera	Melittidae	N	1	1
Melitta leporina	Hymenoptera	Melittidae	N	3	1
Cephus sp.	Hymenoptera	Cephidae	N	2	2
Athalia sp.	Hymenoptera	Tenthredinidae	N	1	1
Chelonus sp.	Hymenoptera	Braconidae	N	1	1
Chrysis sp.	Hymenoptera	Chrysididae	N	3	3
Cerceris sp.	Hymenoptera	Crabronidae	N	10	5
Crossocerus sp.	Hymenoptera	Crabronidae	N	1	1
Lestica sp.	Hymenoptera	Crabronidae	N	4	4
Pemphredon sp.	Hymenoptera	Crabronidae	N	1	1
Philanthus sp.	Hymenoptera	Crabronidae	N	5	4
Gasteruption sp.	Hymenoptera	Gasteruptiidae	N	1	1
Sapyga sp.	Hymenoptera	Sapygidae	N	2	1
Isodontia mexicana	Hymenoptera	Sphecidae	E	4	3
Ancistrocerus sp.	Hymenoptera	Vespidae	N	1	1
Leptochilus sp.	Hymenoptera	Vespidae	N	1	1
Vespa velutina	Hymenoptera	Vespidae	E	1	1
Vespula germanica	Hymenoptera	Vespidae	N	5	5
Cacyreus marshalli	Lepidoptera	Lycaenidae	E	21	13
Lampides boeticus	Lepidoptera	Lycaenidae	N	1	1
Lycaena phlaeas	Lepidoptera	Lycaenidae	N	1	1
Aglais urticae	Lepidoptera	Nymphalidae	N	1	1
Lasiommata maera	Lepidoptera	Nymphalidae	N	1	1
Lasiommata megera	Lepidoptera	Nymphalidae	N	2	2
Pararge aegeria	Lepidoptera	Nymphalidae	N	7	6
Polygonia c-album	Lepidoptera	Nymphalidae	N	1	1
Vanessa atalanta	Lepidoptera	Nymphalidae	N	3	2
Polyommatus icarus	Lepidoptera	Papilionoidae	N	14	9
Pieris rapae	Lepidoptera	Pieridae	N	80	33
Macroglossum stellatarum	Lepidoptera	Sphingidae	N	1	1
